# 非小细胞肺癌胸腔镜与常规开胸术后血清VEGF、MMP-9变化的研究

**DOI:** 10.3779/j.issn.1009-3419.2014.01.04

**Published:** 2014-01-20

**Authors:** 文鑫 田, 宏峰 佟, 耀光 孙, 青峻 吴, 超 马, 鹏 焦

**Affiliations:** 100730 北京，卫生部北京医院胸外科 Department of Thoracic Surgery, Beijing Hospital, Beijing 100730, China

**Keywords:** 肺肿瘤, 胸腔镜, VEGF, MMP-9, Lung neoplasms, Video-assisted thoracoscopic surgery, VEGF, MMP-9

## Abstract

**背景与目的:**

血管内皮生长因子（vascular endothelial growth factor, VEGF）及基质金属蛋白酶-9（matrix metalloproteinase-9, MMP-9）是重要的促血管生成因子，它们在肺癌血管生成中起着非常重要的作用。本研究旨在探讨非小细胞肺癌（non-small cell lung cancer, NSCLC）患者围手术期血清VEGF及MMP-9水平变化的规律，同时比较电视辅助胸腔镜手术（video-assisted thoracoscopic surgery, VATS）及常规开胸手术（traditional open surgery, TOS）后上述指标变化的差异。

**方法:**

选取卫生部北京医院胸外科2010年10月-2012年8月收治的NSCLC患者43例，入组患者均在全麻下行肺叶切除、系统淋巴结清扫术，所有患者均于术前1天、术后第1、2、3、7天抽取静脉血测定血清VEGF及MMP-9的水平，观察围手术期两指标的变化规律。根据手术方式的不同将入组患者分为胸腔镜组（VATS组，25例）及常规开胸组（TOS组，18例），比较两组围手术期血清VEGF及MMP-9变化的差异。

**结果:**

NSCLC患者术后血清VEGF及MMP-9水平均呈先升高后下降的趋势，分别于术后第2天、第3天达到峰值浓度，与术前相比有明显统计学差异（*P*=0.031, *P*=0.020），术后第7天时，两者水平仍高于术前。VATS组和TOS组两组术后VEGF及MMP-9水平也均出现先升高后下降的变化规律，变化幅度以TOS组明显，但两组间两指标变化趋势的差异均无统计学意义（*F*=2.022, *P*=0.163; *F*=1.703, *P*=0.199）。

**结论:**

NSCLC患者术后早期均出现血清VEGF及MMP-9水平的升高，变化幅度以TOS组略明显，但VATS与TOS组间的差异无统计学意义。

## Abstract (en)

**Background and objective:**

Vascular endothelial growth factor (VEGF) and matrix metalloproteinase-9 (MMP-9) are important factors in angiogenesis. These factors function in the angiogenesis of lung cancers. The aim of this study is to determine the changes in serum VEGF and MMP-9 after patients with NSCLC were subjected to lobectomy. The aim of this study is also to compare the differences in the changes observed between video-assisted thoracoscopic surgery (VATS) and traditional open surgery (TOS).

**Methods:**

A total of 43 consecutive patients with NSCLC were recruited from October 2010 to August 2012. All of these patients underwent lobectomy and systematic mediastinal lymphadenectomy. Blood was drawn from these patients to measure the serum levels of VEGF and MMP-9 in the morning before surgery and on postoperative days 1, 2, 3, and 7 (POD1, POD2, POD3, and POD7, respectively). Perioperative variations in serum VEGF and MMP-9 were also observed. Using different surgical options, we divided the patients into two groups: VATS group (25 patients) and TOS group (18 patients). The differences in the changes in serum VEGF and MMP-9 were then compared between the two groups.

**Results:**

After surgery, the serum VEGF and MMP-9 of patients with NSCLC initially increased and subsequently decreased. The peak levels were observed on POD2 and POD3, and these levels were significantly higher than the preoperative levels (*P*=0.031, *P*=0.020). On POD7, the serum VEGF and MMP-9 remained higher than the preoperative levels. In VATS and TOS groups, the serum VEGF and MMP-9 levels also initially increased and subsequently decreased. The TOS group showed a more evident variation. However, no significant statistical differences in the changes of VEGF and MMP-9 were found between the two groups (*F*=2.022, *P*=0.163; *F*=1.703, *P*=0.199).

**Conclusion:**

Serum VEGF and MMP-9 remarkably increase in TOS group and VATS group. Although the serum level of VEGF an MMP-9 in TOS group were higher than that in VATS group. But no significant difference was observed between VATS and TOS group.

血管内皮生长因子（vascular endothelial growth factor, VEGF）及基质金属蛋白酶-9（matrix metalloproteinase-9, MMP-9）在肺癌血管生成中起着非常重要的作用，已有研究^[1-3]^表明，治疗前血清VEGF及MMP-9的水平是非小细胞肺癌（non-small cell lung cancer, NSCLC）患者预测转移、评估预后的良好指标。胸腔镜手术是胸部微创手术的代表，具有创伤小、疼痛轻、恢复快等优势^[4, 5]^，与常规开胸手术（traditional open surgery, TOS）相比，它对促血管生成因子的影响能否也表现出优势还不确定，国内外此方面研究少。本研究旨在通过监测NSCLC患者围手术期VEGF及MMP-9水平的动态变化，探讨NSCLC患者术后早期发生转移的机制，进一步比较电视辅助胸腔镜手术（video-assisted thoracoscopic surgery, VATS）及TOS两种手术方式对上述因子影响的差异，评估VATS手术在上述方面能否表现出优势。

## 1 研究对象和方法

### 1.1 研究对象

2010年10月-2012年8月，卫生部北京医院胸外科住院的NSCLC患者，术前评估无开胸或VATS手术禁忌，拟行肺叶切除、系统性淋巴结清扫手术的患者为待入组者。若手术最终为肺叶切除+系统性淋巴结清扫，且术前术后不存在影响VEGF或MMP-9水平情况的患者为最终入组患者。

入组标准：①肿块 < 6 cm，叶支气管未受侵犯；②行肺叶切除+系统性淋巴结清扫手术；③术前术后不存在影响VEGF或MMP-9水平的情况（合并其他组织或器官的肿瘤、二次手术、脓胸等）；④无远处转移。

根据手术方式不同，入组患者分为胸腔镜手术组（VATS组）和常规开胸手术组（TOS组）。

### 1.2 手术方法

手术中，所有患者均行静脉吸入复合全麻，双腔气管插管，术中均行单肺通气，每个患者均行解剖性肺叶切除+系统性肺门纵隔淋巴结清扫术。

VATS肺叶切除：手术切口由主操作孔、腔镜孔及一个辅助操作孔组成，其中主操作孔切口长约3 cm-5 cm，选用腋前线第4或5肋间进胸，腔镜孔选用腋中线第7或8肋间进胸，辅助操作孔选择肩胛线第7或8肋间。不使用肋骨牵开器，根据情况选用乳突牵开器牵开皮肤肌层或切口保护套。胸内操作完全在镜下完成，支气管、肺裂及较大血管的处理采用一次性切割缝合器，较小血管处理采用丝线或可吸收夹。

TOS肺叶切除：一般选用第5或6肋间后外侧切口进胸，切口长约15 cm-20 cm，胸壁肌肉切断，使用肋骨牵开器牵开肋骨，胸内操作完全为直视下完成，血管、支气管及肺的处理采用切割缝合器，或丝线结扎、缝合。

系统性淋巴结清扫：至少4站淋巴结，其中包括肺门淋巴结在内的肺门肺内淋巴结至少1站，包括隆突下淋巴结在内的纵隔淋巴结至少3站。

### 1.3 材料与仪器

VEGF、MMP-9放射免疫试剂盒，均由北京福瑞生物工程有限公司提供。γ计数器（北京核仪器厂BH6020型组合式γ计数器）、离心机、振荡机、水浴箱、冰箱、微量加样器等。

### 1.4 实验指标测定方法

分别于术前1天，术后第1、2、3、7天清晨，空腹抽取静脉血5 mL，3, 000 r/min离心5 min后取血浆储存于-40 oC冰箱中。待血样收集完成后，分别用放免试剂盒集中测量VEGF、MMP-9的水平。各指标测定时严格按照试剂盒说明书进行操作。

### 1.5 统计学分析

所有数据均以Mean±SD表示，使用软件SPSS 17.0进行数据统计学分析，两组人群资料差异用χ^2^检验或两独立样本t检验，两组间不同时间点VEGF、MMP-9水平差异采用两独立样本t检验或者重复测量数据的方差分析。*P* < 0.05为差异具有统计学意义。

## 2 结果

### 2.1 患者临床及手术资料（表 1）

**1:** VATS组及TOS组患者临床及手术资料 Clinical and surgical characteristics of patients of VATS and TOS group

	VATS group	TOS group	*P*
Numbers	25	18	
Age (yr)	61.56±10.2	61.44±9.8	NS
Gender (M/F)	15/10	10/8	
FEV_1_ (L)	2.49±0.68	2.49±0.61	NS
Tumor diameter (cm)	3.52±1.17	3.82±1.89	NS
Duration of surgery (min)	160.6±48.03	208.1±83.9	NS
Tumor location			
LUL	5	9	
LLL	5	5	
RUL	11	2	
RML	2	0	
RLL	2	2	
Histology			
Adenocarcinoma	18	13	
Squamous cell	6	5	
Large cell	1	0	
TNM stage			
Ⅰ	18	9	
Ⅱ	2	4	
Ⅲa	5	5	
Numbers of lymph nodes dissected	19.6±9.2	16.6±8.6	NS
Groups of lymph nodes dissected	5.16±1.03	4.89±1.13	NS
LUL: left upper lobe; LLL: left lower lobe; RUL: right upper lobe; RML: right middle lobe; RLL: right lower lobe; VATS: video-assisted thoracoscopic surgery; TOS: traditional open surgery; M: Male; F: Female; NS indicates lack of significant differences.			

本研究共43例病例入组，分为VATS组（25例）和TOS组（18例），两组资料见表 1。两组在年龄、性别、术前FEV_1_值、手术时间、肿瘤直径、淋巴结清扫个数、站数等方面无明显统计学差异（*P* > 0.05）。VATS组1例患者术后出现肺漏气，予高渗葡萄糖胸腔内注射，于术后第9天顺利拔除胸管出院；1例于术后第3周左右出现乳糜胸，予以再次开胸手术结扎胸导管后治愈。

### 2.2 围手术期血清VEGF及MMP-9水平的变化（图 1）

**1:**
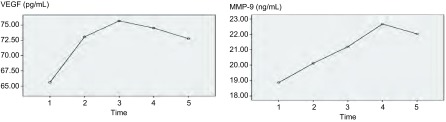
NSCLC患者围手术期血清VEGF及MMP-9水平的变化。Time 1：术前；Time 2：术后第1天；Time 3：术后第2天；Time 4：术后第3天；Time 5：术后第7天。 Changes of perioperative seral VEGF and MMP-9 levels for NSCLC patients. Time 1: the day before surgery; Time 2: the first day after surgery; Time 3: the second day after surgery; Time 4: the third day after surgery; time 5: the seventh day after surgery. VEGF: vascular endothelial growth factor; MMP-9: matrix metalloproteinase-9. NSCLC: non-small cell lung cancer.

将所有入组患者围手术期VEGF及MMP-9水平的变化进行统计学分析。NSCLC患者术后血清VEGF水平呈先升高后下降的趋势，术后第2天达到峰值浓度，并且与术前相比有明显统计学差异（*P*=0.031），到术后第7天时，VEGF水平虽较前下降，但仍高于术前水平。NSCLC患者术后MMP-9水平呈逐渐升高的趋势，于术后第7天时较前略下降。与术前水平比较，术后第3天及第7天MMP-9水平均明显高于术前（*P*=0.020, *P*=0.034）。

### 2.3 VATS组及TOS组围手术期VEGF及MMP-9水平变化的比较（图 2）

**2:**
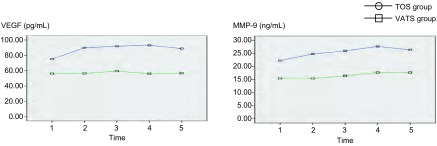
VATS组及TOS组围手术期血清VEGF、MMP-9水平变化的比较。Time 1：术前；Time 2：术后第1天；Time 3：术后第2天；Time 4：术后第3天；Time 5：术后第7天。 Comparison of the changes of perioperative seral VEGF and MMP-9 levels between VATS group and OT group. Time 1: the day before surgery; Time 2: the first day after surgery; Time 3: the second day after surgery; Time 4: the third day after surgery; Time 5: the seventh day after surgery.

按照手术方式不同，将入组患者分为VATS组和TOS组，两组术后VEGF水平均呈现先升高后下降的趋势，变化幅度以TOS组明显，但运用重复测量数据的方差分析，两组间变化趋势差异无统计学意义（*F*=2.022, *P*=0.163）。两组间各时间点VEGF水平比较，均未见明显统计学差异（*P* > 0.05）。VATS组及TOS组术后MMP-9水平均呈现逐渐升高的趋势，以TOS组明显，但两组间变化趋势比较无统计学差异（*F*=1.703, *P*=0.199）。两组间各时间点MMP-9水平比较，均未见明显统计学差异（*P* > 0.05）。

### 2.4 血清VEGF与MMP-9水平的相关性分析

NSCLC患者术前及术后第1、2、3、7天血清VEGF水平与MMP-9水平呈正相关，*Pearson*相关系数分别为0.922、0.910、0.886、0.950、0.978，*P*值均 < 0.05。

## 3 讨论

肿瘤的生长、侵袭和转移必须依赖于血管生成，许多促血管生成因子参与了肿瘤的血管生成过程，其中VEGF及MMP-9在肿瘤的血管生成中起重要作用。VEGF是迄今发现的、最重要的促血管生成因子，它能够调节血管的通透性，促进肿瘤血管生成，许多研究发现在多种肿瘤尤其是转移性肿瘤患者的血清中VEGF水平明显升高，其升高水平与肿瘤转移和复发有关^[6-8]^。MMP-9是MMP家族成员之一，它能够降解细胞外基质，使肿瘤细胞易于突破细胞外基质构成的结构屏障，向周围组织侵袭或出现远处转移。MMP-9的促血管生成作用不仅表现在能够促进具有活性的VEGF的释放^[9]^，还表现在可以直接作为促血管形成因子参与肿瘤新生血管的形成，从而在血管形成方面发挥关键作用^[10]^。

研究^[1-3]^发现，肺癌患者血清中VEGF、MMP-9均有较高水平表达，并且血清VEGF、MMP-9的水平可作为肺癌患者预测转移复发以及评估预后的有效指标。Shimanuki等^[2]^提出血清VEGF浓度与NSCLC患者肿瘤血管生成密切相关，低VEGF水平患者预后明显优于高VEGF水平患者。Laack等^[3]^也得出类似的结论，NSCLC患者治疗前血清VEGF及MMP-9水平与患者预后明确相关，并且血清MMP-9水平可作为一项独立的预后预测因子。另外，NSCLC患者MMPs和肿瘤血管生成这两个系统存在相互协同、相互促进的内在联系。VEGF能上调内皮细胞尿激酶型纤溶酶原激活物，进而激活MMPs，VEGF也能直接诱导细胞MMPs基因的转录，诱导血管内皮细胞产生MMPs。叶晓峰等^[11]^发现NSCLC组织VEGF和MMP-2表达之间存在正相关，我们的研究也分析了血清VEGF与MMP-9水平之间的关系，术前术后各时间点两者均存在明显的正相关（*P* < 0.05），从而提示VEGF和MMP-9在NSCLC的病情演进过程中可能起协同作用，两者共同促进肿瘤的侵袭和转移。

已有研究^[12]^提出，肺癌术后VEGF水平较术前升高，并且进一步动物实验发现VEGF水平的升高能够打破机体在血管生成调节方面的平衡，诱导术后早期肺癌微转移的发生，而应用血管生成抑制剂则能够抑制上述微转移的发生。胡瑛等^[13]^同样发现肺癌原发肿瘤切除后VEGF及MMP-9水平均升高，且VEGF升高的幅度更加明显，与肺良性病患者比较，NSCLC患者术前术后VEGF及MMP-9的水平均明显升高。我们的研究监测了NSCLC术前及术后1、2、3、7天血清VEGF及MMP-9的动态变化，结果发现，NSCLC患者术后上述指标均呈现先升高后下降的变化趋势，分别于术后第2天、第3天达到峰值浓度，术后第7天时较前下降，但仍高于术前水平。我们考虑肺癌术后VEGF及MMP-9水平不降反升的原因有：存在其他的不依赖肿瘤存在产生VEGF及MMP-9的途径；手术操作、挤压、缺氧等因素促进肿瘤细胞扩散；原发灶的切除同时也刺激了残留的微小癌灶加速产生；术后伤口愈合导致促血管生成因子增多等。

我们根据手术方式的不同将入组患者分为VATS组与TOS组，两组术后VEGF及MMP-9水平均呈现先升高后下降的趋势，变化幅度均以TOS组明显，但统计学分析两组间变化的差异无统计学意义（*P* > 0.05）。既往VATS及开胸术后血清VEGF变化比较的研究未见报道，血清MMP-9变化比较的研究也较少，且既往研究结果间存在矛盾。Ng等^[14]^研究认为，VATS及开胸术后MMP-9水平均下降，VATS组MMP-9水平明显低于TOS组（*P* < 0.05）；而Whitson等^[15]^则发现VATS及开胸组术后MMP-9水平先升高后下降，VATS组上升幅度高于开胸组（*P* < 0.05）。我们认为，VATS手术作为已被广泛接受的胸部微创手术，切口小，机体创伤小，术中操作精细，对肿瘤组织挤压、牵拉等影响小，故VATS可能对VEGF、MMP-9的影响也小，但相关研究结论不统一，还需要更深入研究。

综上所述，NSCLC患者术后早期均出现血清VEGF及MMP-9水平的升高，虽然数值上TOS组变化幅度大，但VATS及TOS组间的差异无统计学意义。术后VEGF、MMP-9水平的升高可促进肿瘤新生血管的生成，导致患者术后早期肿瘤的转移、复发。因此，围手术期血清VEGF、MMP-9水平的监测对于评估肺癌预后、研究术后早期转移的机制等有重要意义。
